# Primary isolated ectopic cerebellopontine angle craniopharyngioma: diagnostic considerations, function-oriented resection, and long-term tumor control

**DOI:** 10.3171/2026.7.FOCVID2677

**Published:** 2026-10-01

**Authors:** Ken Matsushima, Michihiro Kohno, Kyosuke Matsunaga, Hiroki Sakamoto, Jiro Akimoto

**Affiliations:** 1 Department of Neurosurgery, Tokyo Medical University, Tokyo; and; 2 Department of Neurosurgery, Kosei Chuo General Hospital, Tokyo, Japan

**Keywords:** cranial nerve preservation, facial nerve monitoring, skull base surgery

## Abstract

Primary isolated ectopic cerebellopontine angle craniopharyngioma is rare and should be considered among cystic cerebellopontine angle lesions. A woman in her 70s presented with a 44-mm lesion extending into Meckel’s cave, with progressive facial nerve palsy, bulbar dysfunction, and gait disturbance. Imaging revealed irregularly enhancing solid components within a homogeneous cyst, peripheral punctate calcifications, and low diffusion signal. Near-total resection was achieved through the retrosigmoid approach under continuous facial nerve monitoring, intentionally leaving adherent capsule. Pathological analysis confirmed adamantinomatous craniopharyngioma. Facial nerve function improved from House-Brackmann grade V to grade II, bulbar dysfunction and gait disturbance resolved, and no regrowth has occurred at 7 years.

The video can be found here: https://stream.cadmore.media/r10.3171/2026.7.FOCVID2677

## Transcript

This video demonstrates the diagnosis and retrosigmoid resection of a primary isolated ectopic cerebellopontine angle craniopharyngioma causing progressive multiple cranial nerve deficits, highlighting monitoring-guided functional preservation and long-term tumor control.

### 0:41 Clinical History.

This lesion had been known for approximately 20 years and had remained radiographically stable for several years before renewed enlargement. The patient, a woman in her 70s, presented with progressive left facial numbness and gait disturbance. She subsequently developed House-Brackmann grade V facial nerve palsy, dysphagia, and hoarseness.

### 1:08 Preoperative Images.

CT demonstrated an approximately isodense, mildly heterogeneous mass in the cerebellopontine angle compressing the brainstem, with focal peripheral punctate calcifications. Contrast-enhanced MRI showed a 44-mm lesion extending into Meckel’s cave, composed of a relatively homogeneous cystic component and irregularly enhancing solid components. The cystic component was hyperintense on both T1- and T2-weighted images and showed low signal on diffusion-weighted imaging.

Schwannoma often contains cystic components, but the calcification is unusual. Meningioma can show calcifications and enhancing solid components, but a predominantly cystic architecture was atypical, as cystic meningiomas are uncommon. Epidermoid cyst can appear predominantly cystic, but the low diffusion signal, rather than diffusion restriction, and enhancing solid components were atypical. Overall, these findings prompted consideration of craniopharyngioma despite the absence of sellar, suprasellar, or pituitary stalk involvement, based on our previous experience with two ectopic cerebellopontine angle craniopharyngiomas.^1–5^

### 2:34 Surgical Procedure.

Given the suggestive imaging findings and progressive cranial nerve deficits, we adopted a function-oriented strategy aimed at maximum safe resection and cranial nerve preservation.^6–8^ A retrosigmoid approach was selected because it avoids temporal lobe retraction and allows early identification of the proximal facial nerve, enabling ball-type electrode placement and continuous, second-by-second monitoring from the early stage of dissection.^9,10^

In the park-bench position, we identified the lower cranial nerves and the facial-vestibulocochlear complex along the dorsocaudal surface of the tumor. After cyst puncture and evacuation of xanthochromic cyst fluid, we obtained a wide working space in the cerebellopontine angle and identified the trigeminal nerve on the cranial aspect of the lesion. After identifying the proximal facial nerve, a ball-type electrode was then placed on the proximal facial nerve to begin serial stimulation monitoring at 1-second intervals. This real-time serial stimulation monitoring helped guide capsule dissection around the facial-vestibulocochlear complex by allowing prompt recognition of amplitude changes.^9,10^ While monitoring real-time electromyographic responses, we removed the solid nodular component behind the facial-vestibulocochlear complex through the space between the facial and vestibulocochlear nerves and the space above the vestibulocochlear nerve. Cranially, the trochlear nerve was also identified, and the solid component near Meckel’s cave was resected. The capsule was followed as far as safely possible while preserving the anterior inferior cerebellar artery and abducens nerve; however, densely adherent portions on the facial-vestibulocochlear complex and brainstem were intentionally left to preserve function. The response amplitude on continuous facial nerve monitoring remained above 90% of baseline throughout the procedure.

### 5:17 Postoperative Course.

Postoperatively, no new neurological deficits developed. Hearing did not deteriorate, and facial nerve function improved from House-Brackmann grade V to grade II. Gait disturbance, hoarseness, and dysphagia resolved. Postoperative contrast-enhanced MRI confirmed near-total resection, with a small residual tumor intentionally left behind the facial-vestibulocochlear complex because of dense adhesion.

Pathology confirmed adamantinomatous craniopharyngioma, characterized by a cyst wall lined by multilayered keratinized squamous epithelium containing wet keratin, with granulation tissue rich in cholesterol clefts and multinucleated giant cells.

No tumor regrowth has been observed, and no additional treatment has been required during 7 years of follow-up.

## Acknowledgments

OpenAI ChatGPT (GPT-5.5 Thinking) was used for language editing and wording refinement of the transcript and slide text, and to assist with minor retouching of an author-created schematic illustration of the monitoring concept. We reviewed and edited all AI-assisted content and take full responsibility for the final submission. AI was not used for data analysis, clinical decision-making, reference generation, or creation/modification of patient-identifiable images or videos.

## Disclosures

The authors report no conflict of interest concerning the materials or methods used in this study or the findings specified in this publication.

## Author Contributions

Primary surgeon: Kohno. Assistant surgeon: Matsushima, Matsunaga, Sakamoto. Editing and drafting the video and abstract: Matsushima. Critically revising the work: Kohno, Matsushima, Akimoto. Reviewed submitted version of the work: all authors. Approved the final version of the work on behalf of all authors: Kohno.

## Supplemental Information

### Patient Informed Consent

The necessary patient informed consent was obtained in this study.
